# Imagining One’s Own and Someone Else’s Body Actions: Dissociation in Anorexia Nervosa

**DOI:** 10.1371/journal.pone.0043241

**Published:** 2012-08-22

**Authors:** Dewi Guardia, Léa Conversy, Renaud Jardri, Gilles Lafargue, Pierre Thomas, Vincent Dodin, Olivier Cottencin, Marion Luyat

**Affiliations:** 1 Laboratoire de Neurosciences Fonctionnelles et Pathologies, EA-4559, Université Lille Nord de France, Lille, France; 2 Service d’Addictologie, Hôpital Calmette, CHRU de Lille, Lille, France; 3 Service de Psychiatrie, Hôpital Fontan, CHRU de Lille, Lille, France; 4 Service de psychiatrie de l’hôpital Saint-Vincent de Paul, Lille, France; 5 Département de Psychologie, Université Lille Nord de France, Villeneuve d’Ascq, France; Royal Holloway, University of London, United Kingdom

## Abstract

**Background:**

Patients with anorexia nervosa (AN) usually report feeling larger than they really are. This body overestimation appears to be related not only to the patient’s body image but also to an abnormal representation of the body in action. In previous work on a body-scaled anticipation task, anorexic patients judged that they could not pass through a door-like aperture even when it was easily wide enough - suggesting the involvement of the body schema. In the present study, we sought to establish whether this erroneous judgment about action is specifically observed when it concerns one’s own body or whether it is symptomatic of a general impairment in perceptual discrimination.

**Methods:**

Twenty-five anorexic participants and 25 control participants were presented with a door-like aperture. They had to judge whether or not the aperture was wide enough for them to pass through (i.e. first-person perspective, 1PP) and for another person present in the testing room to pass through (i.e. third-person perspective, 3PP).

**Results:**

We observed a higher passability ratio (i.e. the critical aperture size to shoulder width ratio) in AN patients for 1PP but not for 3PP. Moreover, the magnitude of the passability ratio was positively correlated not only with the extent of the patient’s body and eating concerns but also with the body weight prior to disease onset. Our results suggest that body overestimation can affect judgments about the capacity for action but only when they concern the patient’s own body. This could be related to impairments of the overall network involved in the emergence of the body schema and in one’s own perspective judgments.

**Conclusion:**

Overestimation of the body schema might occur because the central nervous system has not updated the new, emaciated body, with maintenance of an incorrect representation based on the patient’s pre-AN body dimensions.

## Introduction

Anorexia nervosa (AN) is a serious mental illness that affects 0.5 to 1.0% of women during their lifetime and a much smaller proportion of men [1]. The death rate has been estimated at 5.6% per decade of illness [2]. Anorexic patients usually report feeling fatter and larger than they really are [3]–[5]. This alteration in body representation is a major clinical symptom of AN [6]. It is also a major prognostic factor by increasing body dissatisfaction and the obsessive will to lose weight and thus maintaining restrictive eating behaviours [7]–[10]. Despite the crucial importance of this bias, its exact nature and consequences are poorly understood. At present, body size estimation is assessed by the presentation of body shape drawings correlated with a range of body mass indices [11], [12]. Participants usually have to select the shape that most closely corresponds to their current body. The body mass index (BMI) for the chosen equivalent body shape is then compared with the participant’s actual BMI. The difference between the two values defines the degree of body under- or overestimation. However, these estimates involve different levels of representation and prevent one from distinguishing between (i) the effects of the top-down influences induced by emotions/attitudes towards the body (i.e. the body image) and (ii) disturbances of sensorimotor representation of the body during actions (i.e. the body schema) [13].

Recently, some authors have suggested that the body schema could be disturbed in AN [14], [15]. These distortions may be related to dysfunction of the parietal cortex in general and the right superior parietal lobule in particular [14], [16]–[18], since the latter structure was found to be crucial for establishing a coherent body schema [19]. However, the development of a coherent representation of the body requires prior integration and synthesis of many different sources of sensory information (e.g. visual and proprioceptive information). Even some discrepancies remain between their interpretations, several studies have recently showed disturbance in visuo-tactile [20] and visuo-proprioceptive integration in AN [21].

In one of our previous studies, we tried to investigate more directly the involvement of the body schema. For this purpose, anorexic patients and control participants were asked to judge whether or not an aperture was wide enough for them to pass through [15]. The anticipation of action was found to be severely disturbed in anorexic patients: they judged that they could not pass through an aperture, even when it was easily wide enough. In fact, they behaved as if their body was larger than it really was. This possible overestimation of their body schema may cause a change in the patient’s perception of space. Indeed, the body’s dimensions have a role in the scaling of environmental parameters in extrapersonal space [22]–[25]. Sizes are perceived relative to that of one’s own body [26]. The visual judgment of passability through an aperture was found to be related to an invariant, namely the perceived ratio (Πp, the perceived critical aperture divided by the shoulder width) [23]. Judgments of stepping height have been also shown to be based upon body dimensions, such as leg length [22]. In fact, Warren and Whang [23] found that visual information (e.g. the visual eye-height) was linked to the relevant body part involved in a given action and used to anticipate and perform the action. Our previous results also suggested that in AN the possible neural implementation of the body schema must also be taken into account [15]. However, the results of our experiment raise a new question: do AN patients imagine that other people’s bodies are also larger than they really are or is the impairment specifically linked to overestimation of their own body schema?

A first clue was provided by a recent study evaluating the accuracy of people’s estimates of their own body weight and that of other people [27]. After rating their degree of dietary restraint, participants viewed photographs of 10 women (from underweight to obese) and then estimated their body weight. Restrained and unrestrained eaters did not differ in their estimates of the target’s weight [28], [29]. In contrast, individuals with high dietary restraint underestimated their own weight to a greater extent than those with low dietary restraint [27]. These results strengthen the hypothesis whereby body distortions are related to a specific, subjective bias. It is noteworthy that the participants in these studies were not suffering from eating disorders. This factor might explain why the participants underestimated body weight, in contrast to the body size and weight overestimation observed in patients with AN [3]–[5]. It would also be advisable to replicate these studies in clinical populations.

A second clue was provided by the work of Smeets, Ingleby, Hoek and Panhuysen [29]. The researchers looked at whether individuals with AN visualized themselves as fatter than they really were because they perceived themselves to be fatter. A signal detection analysis showed that the AN and normal and thin control groups did not differ in perceptual sensitivity when judging differences in size between pictures of their own body and pictures of someone else’s body. The researchers did not find significant correlations between body size estimates and perceptual sensitivity and thus concluded that body image abnormalities most probably arise during reconstruction of the visual body image (top-down influences) rather than during perception of the body (bottom-up influences). However, AN patients may show better visual discrimination in the detail-based processing of body forms [30]; the patients were more accurate than controls for discrimination of someone else’s body forms and just as accurate as controls for discrimination of someone else’s body actions. However, as we have seen, these estimates prevent one from distinguishing between (i) the effects of top-down influences (induced by emotions, attitudes and knowledge towards the body, i.e. body image) and (ii) disturbances of the sensorimotor representation of the body (i.e. the body).

In the present study, we sought to establish whether erroneous judgment about action is specifically observed when it concerns one’s own body or whether it is symptomatic of a general impairment in perceptual judgments. In order to test our hypothesis in AN, we added a supplementary condition to the ecological paradigm developed in our previous research [15]. Each participant was required to judge whether or not an aperture was wide enough for them to pass through (i.e. a visuomotor imagery task with a first-person perspective (1PP)) and for another person present in the testing room to pass through (i.e. a third-person perspective (3PP).

Judging passability through an aperture can be considered as a first-person visuomotor imagery task in which perceptual inputs (i.e. aperture width) are interfaced with the motor system, in order to predict the action’s consequences (i.e. passage or failure). This experimental set-up is frequently used to judge the passability of an opening relative to one’s own body size in healthy subjects and in Parkinson’s disease patients [31], [32]. It is now well established that motor imagery and motor execution share kinematic and neural properties [33]–[35]. In healthy subjects, it is thought that the same internal representations are used for motor execution and motor imagery [36], [37]. This similarity is supported by (i) studies showing that mental rehearsal can improve performance [38], (ii) demonstrations of significant overlap between brain areas active in imagined movement and those used for actual movement [39] and (iii) evidence that imagined and executed movements have similar durations and intensities in response to distance and accuracy requirements [35], [40]–[42]. By using gates of different apparent sizes, Decety and Jeannerod [41] reported the same pattern of results for mentally simulated actions. Together with the observed neural overlap between areas activated by imagined actions and those activated by overt actions [34], [43], these results show that motor imagery tasks are both valid and appropriate for assessing body schema integrity [13], [44]. Moreover, mental simulation tasks have been found to be ideal for comparing representations of self with other representations [45]. In order to anticipate their own actions and those of individuals nearby, humans are able to perform various cognitive operations and, in particular, adopt different perspectives (i.e. 1PP and 3PP). Depending on the perspective, these cognitive operations may have either common or differing neural bases [45], [46]. Given our starting hypothesis (i.e. preferential impairment of an individual’s own body actions in the AN group), we expected to see a preferential involvement of 1PP, which might in turn be related to the impairment of a network involving judgments from one’s own perspective and relative to the body schema.

## Methods

### Ethics Statement

This study was approved by an independent ethics committee (Comité de Protection des Personnes Nord Ouest IV; study number: 2007-A01413-50). The study adhered to the tenets of the Declaration of Helsinki. Each participant received a study information sheet and provided her written, informed consent to participation. Parental consent was additionally required for participants under the age of 18.

### Participants

Demographic and clinical data are reported in Table 1. The study included 50 young female participants (25 AN patients recruited from an eating disorder clinic and 25 healthy controls recruited from a student population). The two groups were matched for age and educational level. A psychiatric evaluation of the participants did not reveal any perceptual, attentional or intellectual impairments. The AN patients fulfilled the DSM IV-TR criteria for the disease [6], with 12 restricting types and 13 binge-eating/purging types. Administration of the Mini-International Neuropsychiatric Interview by a psychiatrist confirmed the absence of comorbidities, according to the DSM IV criteria [47], in the two groups. All controls had a normal BMI (i.e. weight/height^2^ ranging from 18.5 to 25 kg/m^2^). Male AN sufferers were not recruited, given their low prevalence and the high rate of psychiatric comorbidities in this population. People with a history of neurological, ophthalmic or bone and joint problems were excluded. Likewise, people receiving psychotropic treatment were excluded from the study.

**Table 1:** Demographical and clinical data for the anorexia nervosa and control groups.

	AN group (n = 25)	Control group (n = 25)	
	Mean (SD)	Mean (SD)	P-value
Age (years)	*23.84 (7.75)*	*24.48 (6.7)*	*0.76^a^*
Educational level	*13.16 (2.56)*	*13.8 (2.814)*	*0.4^a^*
Height (m)	*1.645 (0.061)*	*1.649 (0.059)*	*0.865^b^*
Weight (kg)			
Pre-disease	*54.735 (10.532)*	*NA*	*NA*
6 months prior	*42.890 (7.659)*	*NA*	*NA*
1 month prior	*41.078 (5.913)*	*NA*	*NA*
Current	*42.54 (5.064)*	*60.104 (6.852)*	*<0. 0001^b^*
Current BMI (kg/cm^2^)	*15.645 (1.249)*	*22.06 (2.375)*	*<0.0001^b^*
Shoulder width (cm)	*37.66 (1.553)*	*41.542 (2.493)*	*<0.0001^b^*
BSQ score	*123.96 (33.447)*	*66.708 (17.442)*	*<0.0001^b^*
EDI-2 scores			
Total score	*97.44 (47.836)*	*27.283 (16.505)*	*<0.0001^b^*
DT subscale	*10.4 (5.809)*	*1.583 (2.225)*	*<0.0001^b^*
BD subscale	*14.84 (7.29)*	*8.33 (7.087)*	*0.007^b^*

Educational level: number of years in full-time education after primary school; NA: not applicable; BMI: body mass index; BSE: body size estimation; BSQ: body shape questionnaire; EDI-2: Eating Disorder Inventory, second version; DT: drive for thinness; BD: body dissatisfaction;

(a):T-test;

(b):Mann-Whitney U-test.

### Materials and Procedures

#### Morphological and clinical parameters

The experimenter’s assessments of height, shoulder width and body weight were standardized. Changes over time in nutritional states were measured by considering each patient’s weight at the time of the study and those reported one month and six months before the study. Body dissatisfaction and concerns about weight and shape were assessed in both control and AN groups by administering the Body Shape Questionnaire (BSQ) and the Eating Disorder Inventory-2 (EDI-2), respectively. The BSQ is a one-dimensional, 34-item, self-questionnaire that assesses concerns regarding body shape during the last 4 weeks. Answers are given according to a 6-point Likert scale (i.e. a score of 0 means that the concern is not present and 5 means that it is always present) [48]. The EDI-2 consists of 11 scores measuring psychological features commonly associated with eating disorders [49]. Ninety-one items are rated on Likert scales from 1 (never) to 6 (always). The three following EDI-2 scores were used in the present study: the total score, the “drive for thinness” subscale score and the “body dissatisfaction” subscale score.

#### Simulation of body-scaled action

**Figure 1:**
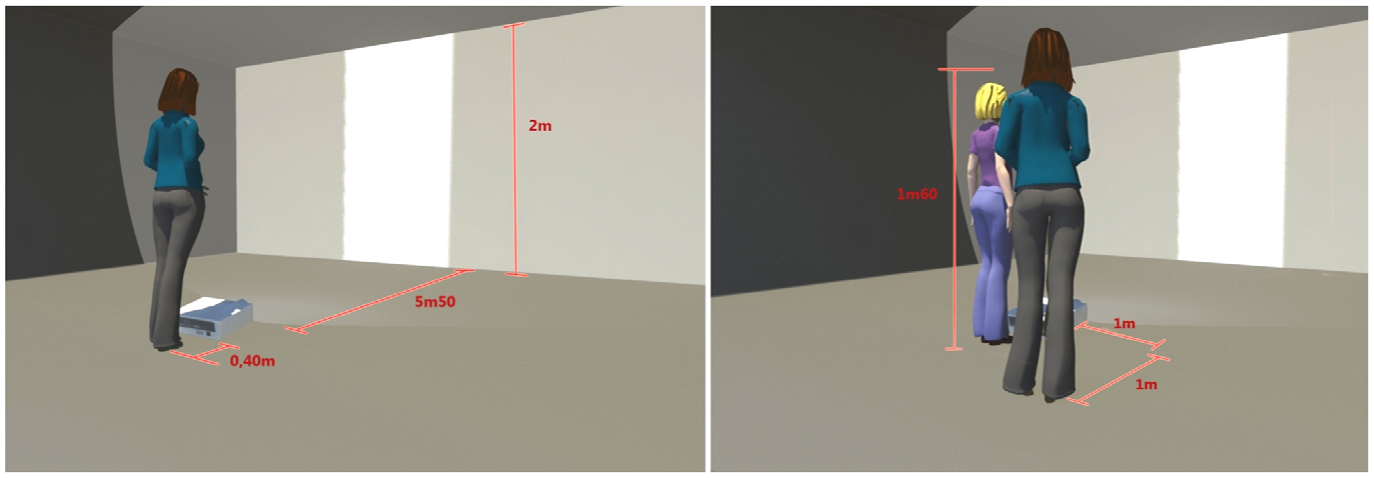
Experimental design, with perspective (third-person-perspective versus first-person-perspective). The distances shown are in metres.

As in our previous study [15], 51 different apertures (varying from 30 cm to 80 cm in width, with a 1 cm increment) were projected onto a wall (in random order and according to a constant-stimulus method) using E-prime software (Psychology Software Tools, Sharpsburg, PA, USA). Each aperture was presented four times. In order to maintain the presentation’s ecological dimensions, the video projector was positioned far enough away from the wall (5.50 m) to allow the projection zone to reach the floor and present a realistic, 2 m-high, door-like aperture. Depending on the experimental conditions, the participant alone or the participant and the experimenter (a 28-year-old women; height: 1.60 m; weight: 52 kg; shoulder width: 38 cm) stood behind the video projector, at a distance of 5.90 m from the wall onto which the aperture was being projected (see Figure 1). Two experimental conditions were tested. In the 1PP condition (see Figure 1a), the participant was instructed to imagine herself walking through the aperture and then to state (i.e. without actually performing the action) whether or not she would be able to walk at a normal speed through the presented aperture without turning sideways. In the 3PP condition (see Figure 1b), the participant was instructed to imagine that the experimenter (standing next to the participant) was going to walk through the aperture. In this condition, the participant was allowed to move and gain a better view of the experimenter, if required. The participant then had to state whether or not the experimenter would have been able to walk through the projected aperture without turning sideways.

For each condition, we determined the perceptual threshold corresponding to the perceived critical aperture, i.e. the aperture for which we obtained a positive (“yes”) response rate of 50%. The perceived critical aperture was calculated as follows: Answer  = 1/[1+ exp(−k(c-aperture))], where ‘c’ is the perceived critical aperture (in cm) with a 50% positive response rate and ‘k’ is the slope of the curve around the point c. For each participant, two perceived passability ratios (Πp) were determined. The ratio for the 1PP condition (Πp_1PP_) was calculated by dividing the perceived critical aperture by the participant’s shoulder width. The ratio for the 3PP condition (Πp_3PP_) was calculated by dividing the perceived critical aperture by the experimenter’s shoulder width. The slope of the psychometric curve provided information on the discriminability of the performance. A difference in slope between the groups would thus reflect a difference in discrimination. It must be noted that a ratio equal to 1 means that the perceived critical aperture is equal to the shoulder width; this corresponds to a strong risk of collision with the sides of the aperture if the action is actually performed. Consequently, a ratio higher than 1 can be interpreted as a safety margin. However, if one considers the situation of two subjects (subject A, with a critical aperture of 40 cm and a shoulder width of 50 cm, i.e. ratio of 0.80), and subject B, with a critical aperture of 50 cm and a shoulder width of 40 cm, i.e. ratio of 1.25), it can be seen that the deviation of the ratio’s value from 1 is not the same in cases of underestimation (ranging from 0 to 1) and cases of overestimation (ranging from 1 to infinity). To avoid artefacts related to the use of this ratio, we subsequently evaluated the judgment of passability by calculating a second metric (the percentage overestimation) as follows: Answer  = [100*(critical aperture-shoulder width)]/shoulder width.

### Statistical Analysis

All analyses were performed with Statistica 7.1 software (Statsoft Inc., Tulsa, OK, 2007). To study the respective influences of group and perspective, an analysis of variance (ANOVA) with repeated measures was performed on the mean perceptual ratios in both conditions and with group as the categorical predictor. A post-hoc comparison was performed with a Newman-Keuls test. The validity of each test’s conditions of application was always determined beforehand. Non-parametric Mann-Whitney and Spearman tests were used when non-normal distributions and non-homogenous inter-group variances were observed.

## Results

The two groups were well matched in terms of morphological and clinical parameters (see Table 1). Thus, there were no significant inter-group differences in age (mean_AN_ ±SD: 23.84±7.75 *vs.* mean_C_: 24.48±6.7; t_48_ = −0.31, p = 0.76), educational level (mean_AN_: 13.16±2.56 *vs.* mean_C_: 13.8±2.814; t_48_ = −0.84, p = 0.40) or height (median_AN_: 1.64, median_C_: 1.662; U = 291.5, Z = −0.17, p = 0.865). By contrast, the mean BMI was 15.65±1.25 for AN patients and 22.06±2.37 for controls: U = 0, Z = −6, p<0.0001. Median shoulder width was also significantly greater in the control group (42 cm) than in the AN group (37.5 cm; U = 73, Z = −4.527, p<0.0001), reflecting the patients’ state of malnutrition. Changes in the patients’ nutritional states were characterized by an average weight gain over the previous month of 1.074 kg±2.884 and an average weight loss over the last 6 months of 0.628 kg±5.234. Unfortunately, the interview data did not specify weight changes over the last six months for five patients. The mean time since disease onset was 4.33±3.57 years. When compared with the control group, the AN group had a significantly higher EDI-2 total score (median_AN_: 96, median_C_: 27; U = 66, Z = 4.668, p<0.0001) and subscales (“search for thinness”; median_AN_: 11; median_C_: 0.5, U = 65, Z = 4.688, p<0.0001; “body dissatisfaction”; median_AN_: 14, median_C_: 6.5; U = 165, Z = 2.674, p = 0.007). The BSQ scores were also significantly greater in the AN group than in the control group (median_AN_: 115, median_C_: 64; U = 36.5, Z = 5.259, p<0.0001).

**Figure 2:**
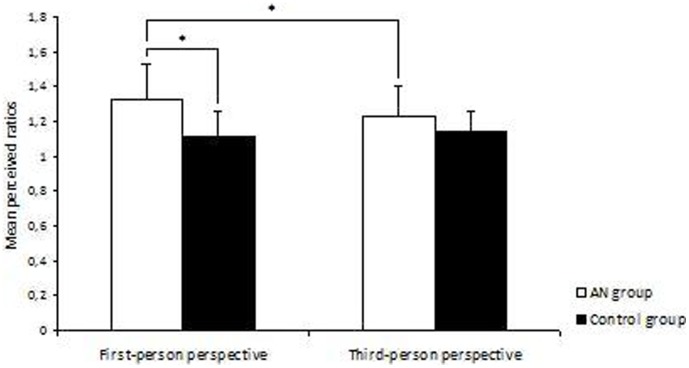
Passability ratios for AN and control groups during first-person-perspective body action or third-person-perspective body action. The perceived passability ratios were calculated by dividing the perceived critical aperture (in centimetres) by the participant’s shoulder width (in centimetres) for the 1PP condition and dividing the perceived critical aperture by the experimenter’s shoulder width for the 3PP condition. The brackets indicate the confidence interval for the mean values. Significant differences (p<0.05) are indicated by*.

The results for the body-scaled action-anticipation tasks are summarized in Table 2 and Figure 2. In the 1PP condition, the mean perceptual ratios (∏p_1PP_) were significantly higher in the AN group than in the control group: mean_AN_: 1.321±0.255; mean_C_: 1.106±0.19; t_48_ = 3.389, p = 0.001. In contrast, the two groups’ mean perceptual ratios (∏p_3PP_) did not different in the 3PP condition (see Figure 2 and Table 2). Although the average ratio in the AN group was slightly higher than that of the control group, this difference was not statistically significant: mean_AN_: 1.227±0.22; mean_C:_ 1.128±0.154, t_48_ = 1.137, p = 0.101. A 2 (group)×2 (condition) ANOVA with repeated measures on “condition” factor and “group” as the categorical predictor for the Πp ratio revealed a significant effect of group: F_1,48_ = 8.653, p = 0.005. In contrast, there was no significant effect of condition: F_1,48_ = 1.316, p = 0.257. However, the interaction between group and condition was statistically significant: F_1,48_ = 4.978, p = 0.03. A post-hoc comparison showed a significant difference between the 1PP and 3PP conditions in AN patients: mean_1PP_: 1.321±0.255 versus mean_3PP_: 1.227±0.22; Q_48_ = 1.2269, p = 0.02. The difference was not significant in controls: mean_1PP_: 1.106±0.19; mean_3PP_: 1.128±0.154; Q_48_ = 1.1367, p = 0.447. Lastly, a statistical analysis did not reveal a significant effect of the clinical subtype (restricting vs. binge-eating/purging types) in any of the conditions (all p>0.1, non-significant).

**Table 2:** Slope, critical aperture and perceived ratio as a function of participant group and perspective.

	AN group (n = 25)		Control group (n = 25)	
	Mean (SD)	Median (Min; Max)	Mean (SD)	Median (Min; Max)
Slope				
1PP	*−0.691 (0.816)*	*−0.476 (−4.439; −0.236)*	*−0.541 (0.179)*	*−0.518 (−0.922; −0.163)*
3PP	*−1.122 (2.919)*	*−0.459 (−15.084; −0.274)*	*−1.128 (2.497)*	*−0.589 (−13.028; −0.249)*
Critical aperture				
1PP	*49.774 (10.1)*	*49.273 (34.478; 70.94)*	*45.964 (7.383)*	*44.446 (33.787; 62.75)*
3PP	*48.462 (8.693)*	*46.77 (34.282; 68.58)*	*44.898 (6.15)*	*44.839 (33.954; 58.381)*
Ratio				
1PP	*1.321 (0.255)*	*1.294 (0.895; 1.87)*	*1.106 (0.187)*	*1.062 (0.795; 1.569)*
3PP	*1.227 (0.22)*	*1.184 (0.868; 1.736)*	*1.137 (0.156)*	*1.135 (0.859; 1.478)*
Percentage overestimation				
1PP	*32.086 (25.483)*	*29.436 (−10.445; 86.986)*	*10.65 (18.735)*	*6.251 (−20.501; 56.875)*
3PP	*22.689 (22.008)*	*18.405 (−13.209; 73.62)*	*13.666 (15.569)*	*13.517 (−14.041; 47.801)*

1PP: first-person perspective; 3PP: third-person perspective.

In order to check that the inter-group difference was not due to a difference in perceptual discrimination, we analysed the slopes of the psychometric curves (see Table 2). There were no significant inter-group differences in discriminability in either the 1PP condition (t_48_ = −0.8998, p = 0.373) or the 3PP condition (t_48_ = 0.0086, p = 0.993).

We also analysed the percentage overestimation (see Table 2), a 2 (group)×2 (condition) ANOVA with repeated measures on the “condition” factor and “group” as the categorical predictor revealed a significant effect of group: F_1,48_ = 8.653, p = 0.005. In contrast, condition did not have a significant effect: F_1,48_ = 1.316, p = 0.257. However, the interaction between group and condition was statistically significant: F_1,48_ = 4.978, p = 0.03. According to the post-hoc analysis, the mean percentage overestimation in the 1PP condition was significantly higher in the AN group than in the control group: mean_AN_: 32.086±25.483; mean_C_: 10.65±18.735; Q_48_ = 10.65, p = 0.026. In the 3PP condition, the mean percentage overestimation was also higher in the AN group than in the control group but the difference was not statistically significant: mean_AN_: 22.689±22.008; mean_C_: 13.666±15.569; Q_48_ = 1.673, p = 0.224. Lastly, the difference between the 1PP and 3PP conditions was significant in AN group (mean_1PP_: 32.086±25.483 versus mean_3PP_: 22.689±22.008; Q_48_ = 32.086, p = 0.021) but not in the control group (mean_1PP_: 10.65±18.735; mean_3PP_: 13.666±15.569; Q_48_ = 13.666, p = 0.447).

Calculation of Spearman’s correlation coefficient (ρ) confirmed the significance of the relationship between the 1PP body-scaled action-anticipation task on one hand and body concerns and eating disorders on the other. There were significant, positive correlations between ∏p_1PP_ and the BSQ score (ρ = 0.548, t_47_ = 4.487, p<0.0001), between ∏p_1PP_ and the EDI-2 total score (ρ = 0.457, t_47_ = 3.519, p = 0.0009), between ∏p_1PP_ and the EDI-2 “drive for thinness” subscale (ρ = 0.487, t_47_ = 3.824, p = 0.0003) and between ∏p_1PP_ and the EDI-2 “body dissatisfaction” subscale (ρ = 0.547, t_47_ = 4.480, p<0.0001). Moreover, our analysis did not reveal a significant correlation between the height and perceived ratios for either the ∏p_1PP_ condition (ρ = 0.064, t_48_ = 0.443, p = 0.659) or the ∏p_3PP_ condition (ρ = −0.06, t_48_ = −0.416, p = 0.679). In the AN group, the ∏p_1PP_ was not related to variations in the BMI over the previous month (ρ = −0.0448, t_21_ = −0.2, p = 0.422; one-tailed test) or the six last months (ρ = −0.236, t_47_ = −1.06, p = 0.151; one-tailed test). However, ∏p_1PP_ was related to the patient’s body weight before disease (ρ = 0.379, t_18_ = 1.738, p = 0.049; one-tailed test) for the whole AN group. The heterogeneity in weight changes during the previous month are related to the timely provision of nutritional support, which varies from one patient to another. Indeed, some of patients in the AN group were in renutrition, whereas some others were still in an undernutrition phase. An analysis of the subgroup of patients in the undernutrition phase revealed a correlation between performance and weight loss over the last 6 months: ρ = 0.644; t_8_ = 2.383, p = 0.022 (one-tailed test). However, analysis of this subgroup did not reveal significant correlations between the 3PP behavioural data on one hand and the EDI-2 subscale, BSQ or anthropometric data (such as BMI) on the other (all p>0.1, non-significant).

## Discussion

The present study investigated the everyday human ability to make judgments about one’s own and other people’s body-scaled actions and then sought to clarify to what extent this ability is disrupted in AN. We confirmed previous results [15] showing that a population of AN patients significantly overestimated their own passability (relative to a control group) in a simulated body-scaled action. These data were concordant with the patients’ clinical complaints that they feel larger than they really are. Another key finding was that passability ratios in AN patients were significantly affected in the 1PP condition but not in the 3PP condition. In contrast, the control group’s judgments of 1PP and 3PP passability were not different. The performance differences between the AN and control groups were not likely due to worse discrimination of visual stimuli by the patients, since there was no statistically difference between the slopes of the respective psychometric curves. Moreover, another factor could explain the augmented ratios in AN: the visual eye-height. However, the mean height was not different in the two groups and thus an eye-height effect could not explain the observed differences in judgement. To control for the participant’s eye-height, we also took care to ensure that this parameter did not change during the experimental session (the participant always stood upright). These overall results suggest that the overestimation of the passability ratios in AN are likely to be caused by an overestimation of their own body schema. They are not symptomatic of a general impairment in perceptual judgments. The fact that the perceived passability ratio (thought to be invariant) was in fact augmented in AN prompts us to suggest that even though the visual eye-height has been shown to be a critical parameter in body-scaled actions [23], [25], the representation of the body in action, naming the body schema, must also be taken into account. If the body schema is distorted, it could affect simulated body-scaled actions.

This overestimation of the body schema in AN can be related to the existence of disturbance in multisensory integration in AN [20], [21], since the body schema is the product of multisensory integration of visual, tactile, proprioceptive and vestibular inputs. For instance, Case, Wilson and Ramachandran have recently shown a deficit in visuo-tactile integration by comparing the strength of the well-known size-weight illusion (SWI) in individuals suffering from AN [20]. A SWI arises when two objects of equal weight but different sizes are weighed. Participants consistently estimate the smaller as being heavier [50]. The illusion is due to the integration of conflicting visual and tactile perceptions. In fact, Case et al. found that AN patients are less susceptible to the SWI than controls and thus suggested that the integration of visual and proprioceptive information could be impaired in AN [20]. This deficit in multisensory integration in AN [16], [51] could be at the origin of the overestimation of the body schema. Moreover, Keizer et al. [52] have shown that AN patients overestimated distances between tactile stimuli applied to the arm and the abdomen suggesting that the tactile body image would be larger than in reality. According to the authors, overestimation of the tactile body image and preferential processing of proprioceptive information would explain why AN patients feel bigger and heavier than they actually are.

However, another research has recently shown that AN patients had a stronger rubber hand illusion (RHI) [21]. In the RHI [53], participants view a dummy hand being stroked with a paintbrush. At the same time, the experimenter applies identical brushstrokes to the participant’s own hand, which is out of the participant’s view. If this visual and tactile information are applied synchronously and if the visual appearance and position of the dummy hand is similar to the participant’s own hand, then some people may feel that the stimuli are coming from the dummy hand and even that the latter is, in some way, part of their own body. This phenomenon requires multisensory integration, which is known to be disrupted in AN, but also the dominance of visual information about hand location on proprioceptive information. Eshkevari et al. suggested that AN patients could preferentially rely on visual information [21]. This apparent contradiction with Case et al.’ results requires to be further clarified in future research. As a suggestion for future investigation, De Vignemont [13] proposed a dynamic, Bayesian approach to body representation which focuses on task’s specificity and demands (e.g. action-oriented tasks versus non-action oriented tasks) and which goes beyond mere bottom-up and top-down views of body representations.

Our correlation analysis confirmed the relationship between the 1PP body-scaled action-anticipation task and the severity of eating disorders by revealing a significant, positive correlation between the patient’s own body action on one hand and body concern, body dissatisfaction and drive for thinness on the other. This disruption may cause restrictive eating behaviours to persist [10], as evidenced by a significant correlation between motor imagery performance levels and prognostic factor such as the EDI-2 “drive for thinness” subscale [8]. Nutritional states, body size and weight changes in AN which constitute potential sources of bias because malnutrition could lead to the impairment of sensory integration and/or changes in body size. Indeed, the body’s dimensions have a role in the scaling of environmental parameters in extrapersonal space [22]–[25]. Apparent sizes are perceived relative to both the visual eye-height [23], [25] and the size of one’s body [24], [26]. However the body size is profoundly modified during decompensation phases. The patients’ 1PP performances were not related to their BMI variations over the previous month or the previous six months but were related to their pre-AN body weight. Our analysis of a subgroup of patients having lost weight over the previous 6 months revealed a positive correlation between the motor imagery performance and body weight loss; the greater the weight loss, the greater the perceived passability ratio. This finding provides a possible explanation for the disruption of body-scaled actions in anorexic people: the body schema modified by the rapid weight loss may not have been updated by the central nervous system [15], [20]. Anorexia nervosa mainly affects young women in the 15–19 age group [54], [55]. However, many of the neurological, morphological and psychological changes occur during puberty and they will have an impact on the body schema. Weight changes induced by eating disorders could enhance these disturbances. The knowledge gained by studying neurological phenomena such as phantom limbs might shed light on this topic. In fact, many amputees continue to feel the presence of a phantom limb after amputation [56], [57]. Many explanatory models of phantom limb syndrome have emerged in recent years. One of these postulates a degree of mismatch between the sensory feedback from the phantom and the cortical regions representing the limb [58]. In anorexic patients, there could be a conflict between the previous body schema (i.e. before the weight change) and the current sensorimotor feedback. As noted by Riva in his allocentric lock hypothesis [59], *“our spatial experience, including the bodily one, involves the integration of different sensory inputs within two different reference frames egocentric (body as reference of first-person experience) and allocentric (body as object in the physical world). (…) They influence each other during the interaction between long- and short-term memory processes in spatial cognition. If, for some reasons, this process is impaired, the egocentric sensory inputs are no more able to update the contents of the allocentric representation of the body: the subject is locked to it”*. Thus, patients would find themselves locked into a larger body. As mentioned above, body dimensions have a role in the scaling of environmental parameters in extrapersonal space [24], [26]. In AN, this scaling may be based on obsolete physical dimensions.

However, there are several limitations to our research. Firstly, the body schema may not be impervious to top-down influences. Indeed, recent data show that psychosocial factors affect visual perception. Morgado et al. [60] investigated the relationship between affective closeness and the perception of passing through the gap between two people. People feel discomfort when they are near to someone to whom they are not affectively close. In Morgado et al.’s study, participants had to imagine passing through the aperture between two life-size pictures of classmates. The authors found that the closer participants felt to their classmates, the more they felt able to pass between them; affective representations indeed had an influence. Since our experimental paradigm involved the judgment of passability through an opening relative to one’s own body size, it should have limited top-down influences.

Secondly, another limitation of the present study relates to the small sample size, as indicated by the degrees of freedom in the statistical analysis (n = 20 for the body weight variation analysis and n = 10 for decompensated phase). Furthermore, the history of weight changes was not reported by all the enrolled patients. Replication of these results in a larger sample (particularly during the undernutrition phase) is now required. Particular attention should be paid to the time since disease onset and the number of decompensations, since it is likely that these two variables influence the change over time in body representations and add to the risk of recurrence. Indeed, we would expect the 1PP vs. 3PP difference to be at its largest during decompensation. Another study limitation relates to the 3PP condition; during this task, the experimenter is likely to be closer to controls than to anorexic patients in terms of body weight and body size. The question should really be whether or not anorexic patients make similarly erroneous judgments about another person with the same body size. However, this would probably involve patients make judgments about another patient, which is ethically problematical and may compromise confidentiality.

Thirdly, the passability estimate does not provide a true visuomotor measurement of the width at which participants would actually begin to turn sideways in order to walk through the aperture. Although motor imagery tasks are valuable and appropriate for assessing body schema integrity [13], [44], it would be informative to film AN sufferers when they really pass through different apertures and encode the aperture for which they begin to turn sideways. We are already planning to perform this type of study, with a view to confirming similar patient behaviours in simulation and in action.

In overall, our findings suggest that the anticipation of body-scaled actions can be strongly disturbed in AN. The body schema overestimation bias observed in AN seems solely related to the patient’s own body action and may be related to specific brain regions involved in the body schema but perhaps also on brains regions underlying judgments at 1PP. A mismatch between the actual sensory feedback and the cortical regions representing the body may lead to the maintenance of an incorrect body representation. Future research and therapeutic approaches (especially virtual reality, for example) could focus on these targets.

Neuroimaging studies might be able to highlight the neural basis of this type of dysfunction. Motor imagery and movement execution have a number of common kinematic and neural properties [33]–[35]. Moreover, mental simulation tasks have been shown to be ideal for comparing representations of self and other representations [45]. In order to anticipate their own actions and those of individuals nearby, humans are able to perform various cognitive operations and, in particular, adopt different perspectives (i.e. 1PP and 3PP). Depending on the perspective, these cognitive operations may have either common or differing neural bases [45], [46]. The common network for 3PP and 1PP anticipation includes the supplementary motor area, the precentral gyrus and the precuneus. In contrast, 3PP-specific neural activity has been found in the right inferior parietal lobule, the posterior cingulate and the frontopolar cortex, whereas 1PP activities recruited the left inferior parietal lobule and the left somatosensory cortex [45]. On the basis of our preliminary results (i.e. preferential impairment of the participant’s own body actions in the AN group), we would expect to see preferential involvement of 1PP, which in turn could be related impairment of a network involving the left inferior parietal lobule and the left somatosensory cortex (involved in one’s own perspective judgments) and right parietal cortex (involved in the body schema).

Over the last decade, virtual reality has emerged as a technology that is especially suitable not only for the assessment of body image disturbances but also for its treatment [61]. Several virtual environment-based software systems have already been developed for this purpose [62], [63]. We believe that the full-body illusion paradigm could be an interesting additional tool [64]. Indeed, Normand, Giannopoulos, Spanlang, and Slater recently used an immersive virtual reality paradigm to study this conflict in healthy subjects [65]. The latter authors showed that the combination of 1PP immersion in a virtual body with synchronous multisensory stimulation could temporarily produce changes in body representation (a larger belly size, in fact). Experiencing this type of 1PP effect in an immersive environment might treat the body distortions experienced by anorexic patients and help them develop a more realistic perception of their own body.
